# International travel as a risk factor for gastrointestinal infections in residents of North East England

**DOI:** 10.1017/S0950268824000827

**Published:** 2024-05-27

**Authors:** Nicola K. Love, Claire Jenkins, Noel McCarthy, Kate S. Baker, Petra Manley, Deborah Wilson

**Affiliations:** 1 National Institute for Health Research Health Protection Research Unit (NIHR HPRU) in Gastrointestinal Infections, University of Liverpool, Liverpool, UK; 2 Field Services, Health Protection Operations, UK Health Security Agency, Newcastle upon Tyne, UK; 3 Gastrointestinal Bacteria Reference Unit, UK Health Security Agency, London, UK; 4 Institute of Population Health, Trinity College Dublin, Ireland; 5 Department for Clinical Infection, Microbiology, and Immunology, University of Liverpool, Liverpool, UK; 6 Department of Genetics, University of Cambridge, Cambridge, UK; 7 North East Health Protection Team, UK Health Security Agency, London, UK

**Keywords:** gastrointestinal infections, travel, infection, epidemiology, surveillance

## Abstract

International travel is thought to be a major risk factor for developing gastrointestinal (GI) illness for UK residents. Here, we present an analysis of routine laboratory and exposure surveillance data from North East (NE) England, describing the destination-specific contribution that international travel makes to the regional burden of GI infection.

Laboratory reports of common notifiable enteric infections were linked to exposure data for cases reported between 1 January 2013 and 31 December 2022. Demographic characteristics of cases were described, and rates per 100,000 visits were determined using published estimates of overseas visits from the Office for National Statistics (ONS) International Passenger Survey (IPS).

About 34.9% of cases reported international travel during their incubation period between 2013 and 2022, although travel-associated cases were significantly reduced (>80%) during the COVID-19 pandemic. Between 2013 and 2019, half of *Shigella spp.* and non-typhoidal *Salmonella* infections and a third of *Giardia sp.*, *Cryptosporidium spp.*, and Shiga toxin-producing *Escherichia coli* (STEC) infections were reported following travel. Rates of illness were highest in travellers returning from Africa and Asia (107.8 and 61.1 per 100,000 visits), with high rates also associated with tourist resorts like Turkey, Egypt, and the Dominican Republic (386.4–147.9 per 100,000 visits).

International travel is a major risk factor for the development of GI infections. High rates of illness were reported following travel to both destinations, which are typically regarded as high-risk and common tourist resorts. This work highlights the need to better understand risks while travelling to support the implementation of guidance and control measures to reduce the burden of illness in returning travellers.

## Introduction

Gastroenteritis is a common cause of morbidity, with estimates suggesting up to 17 million cases annually in the UK [1]. While many cases are relatively mild and short-lived, particularly those caused by viral pathogens such as norovirus, others can result in more prolonged or severe illness and may require hospitalization or lead to death. Bacterial and parasitic pathogens, which are more commonly associated with severe outcomes, are usually acquired through foodborne or waterborne routes, as opposed to viral pathogens, which are generally acquired through person-to-person transmission [2]. In high-income countries, international travel is thought to be a major risk factor for gastrointestinal (GI) illness, particularly for bacterial and parasitic pathogens. Risk is often associated with destination country, with pathogens often endemic in lower- to middle-income (LMIC) destination countries, where sanitation and hygiene are more often compromised.

Estimates suggest that up to 60% of international travellers will develop diarrhoea [3, 4], with morbidity highest in those visiting LMICs. However, many studies are conducted within travel clinic settings, which may bias findings towards travellers at greater risk of developing illness due to the nature of their travel plans. The incidence of GI illness associated with travel is thought to have decreased over the last 20 years, particularly in travellers to countries that were previously high risk but have seen considerable economic improvement, such as areas of East Asia and South America [5]. However, GI illnesses remain one of the most common health complaints reported by travellers, with areas such as South Asia and Africa consistently reported as being associated with a higher risk of illness [3].

While the destination of travel is thought to be the biggest risk factor, other factors influence the likelihood of developing a GI illness while travelling. These factors include activities thought to be higher-risk including types of travel such as backpacking and visiting friends and family, and food choices taken [6]. In addition, certain groups have been shown to have increased susceptibility, including individuals at extremes of age, those with immunosuppression, and those with GI conditions such as inflammatory bowel disease [5]. Furthermore, international travel is a known risk factor for the acquisition of resistant organisms into the gut microbiota. Studies have shown that a higher proportion of multidrug-resistant GI pathogens are isolated from patients reporting recent travel outside the UK [7, 8].

Having a better understanding of travel-associated enteric pathogens could help to improve pre-travel guidance and support public health actions, which could ultimately lead to a reduction in travel-associated GI infections and, potentially, the importation of antimicrobial resistance (AMR), and a reduction in the overall burden of GI infections in settings such as the UK. In England, all laboratory-confirmed cases of notifiable enteric infections are reported to the United Kingdom Health Security Agency (UKHSA) from all National Health Service (NHS) laboratories via England’s main infectious disease laboratory surveillance system, the Second-Generation Surveillance System (SGSS). North East (NE) England is unique in that it has its own surveillance system, EpiNorth3, which links routinely collected SGSS data, laboratory typing data, and exposure data from standardized exposure questionnaires. Here, we describe the epidemiology of GI infections in residents of NE England providing insight into the contribution that international travel makes to the overall and pathogen-specific burden of GI infection in the region.

## Methods

### Definitions and exclusions

Exposure questionnaires are undertaken with all NE residents notified with laboratory-confirmed *Cryptosporidium* spp., *Giardia* sp., hepatitis A, *Salmonella* spp. (typhoidal and non-typhoidal), *Shigella* spp., Shiga toxin-producing *Escherichia coli* (STEC; O157 and certain non-O157 serotypes), *Vibrio* spp., and *Yersinia* spp. infections. Campylobacteriosis cases are excluded from this study as exposure questionnaires are not routinely performed. Listeriosis cases were also excluded from this study to avoid deductive disclosure due to low numbers.

Data on enteric infections reported to UKHSA between 1 January 2013 and 31 December 2022 were extracted from EpiNorth3 in January 2023. Cases were defined as being associated with international travel if the case had a completed exposure questionnaire and reported travel outside of the UK during the standardized incubation period specified in the exposure questionnaire (7 days prior to onset: non-typhoidal *Salmonella* spp., *Shigella* spp., STEC, and *Yersinia* spp.; 14 days prior to onset: *Cryptosporidium* spp. and *Giardia* sp.; 60 days prior to onset: typhoidal *Salmonella* spp.; and 8 weeks prior to onset: hepatitis A). UK-acquired cases were defined as cases with a completed exposure questionnaire who did not report travel outside of the UK during the standardized incubation period. Cases without an exposure questionnaire were defined as having an unknown travel status and were excluded from analyses unless otherwise stated. Given the reduction in international travel reported in England during 2020 and 2021 as a result of the COVID-19 pandemic response, cases reported in 2020 and 2021 (pandemic years) were also excluded from analyses unless otherwise stated.

### Analysis

All analyses were performed using RStudio version 4.2.0. Demographic data including ethnicity, sex, and age were extracted from EpiNorth3. Deprivation and urban–rural classification of residence were derived from the postcode of residence recorded in EpiNorth3 using the publicly available English indices of deprivation 2019 data set [9] and the 2011 rural–urban classification (RUC2011) data set [10]. Directly standardized rates of illness per 100,000 population were calculated for age and ethnic group with denominator data on the NE England population taken from the 2021 census and 2021 mid-year population estimates [11], with 95% confidence intervals (CIs) calculated using the Dobson method. Chi-squared tests were performed for categorical variables.

Destination countries reported in exposure questionnaires were extracted from EpiNorth3. Destinations reported as resorts or cities and incorrectly spelt destinations were recoded. Where multiple locations were recorded during an incubation period, the location was recoded to ‘Multiple/unspecified’. Countries were recoded based on nomenclature used in the UK Office for National Statistics (ONS) International Passenger Survey (IPS) Travelpac data set, to account for sovereignty [12]. Within the EpiNorth3 data set, there was no distinction between Northern Cyprus and the Republic of Cyprus; therefore, both are reported as Cyprus.

Using published estimates from the ONS IPS, it was possible to establish the most common travel destinations for NE England residents. Using visits as a denominator, rates of illness were determined by destination. Countries were grouped into global regions (Africa, Asia, America and Caribbean, Europe, the Middle East, and the Rest of World) as specified in the Travelpac data set. Rates per 100,000 visits were calculated using the total number of visits to each country or country group between 2013 and 2019 calculated using the ‘Final weight’ variable in the Travelpac data set for 2013–2019 and the total number of cases reporting travel to the location between 2013 and 2019. At the time of analysis, Travelpac data were unavailable for 2020–2022.

## Results

Between 2013 and 2022, 9,358 laboratory-confirmed cases of GI illness resulting from infection with *Cryptosporidium* spp., *Giardia* sp., hepatitis A, *Salmonella* spp. (typhoidal and non-typhoidal), *Shigella* spp., Shiga toxin-producing *E. coli* (STEC; O157 and certain non-O157 serotypes), *Vibrio* spp., and *Yersinia* spp. were reported in NE England residents. Routine exposure questionnaires were completed for 7,909 cases (84.5%), of which 2,764 cases (34.9%) reported international travel during their incubation period.

### Travel as a risk factor over time

**Figure 1.**
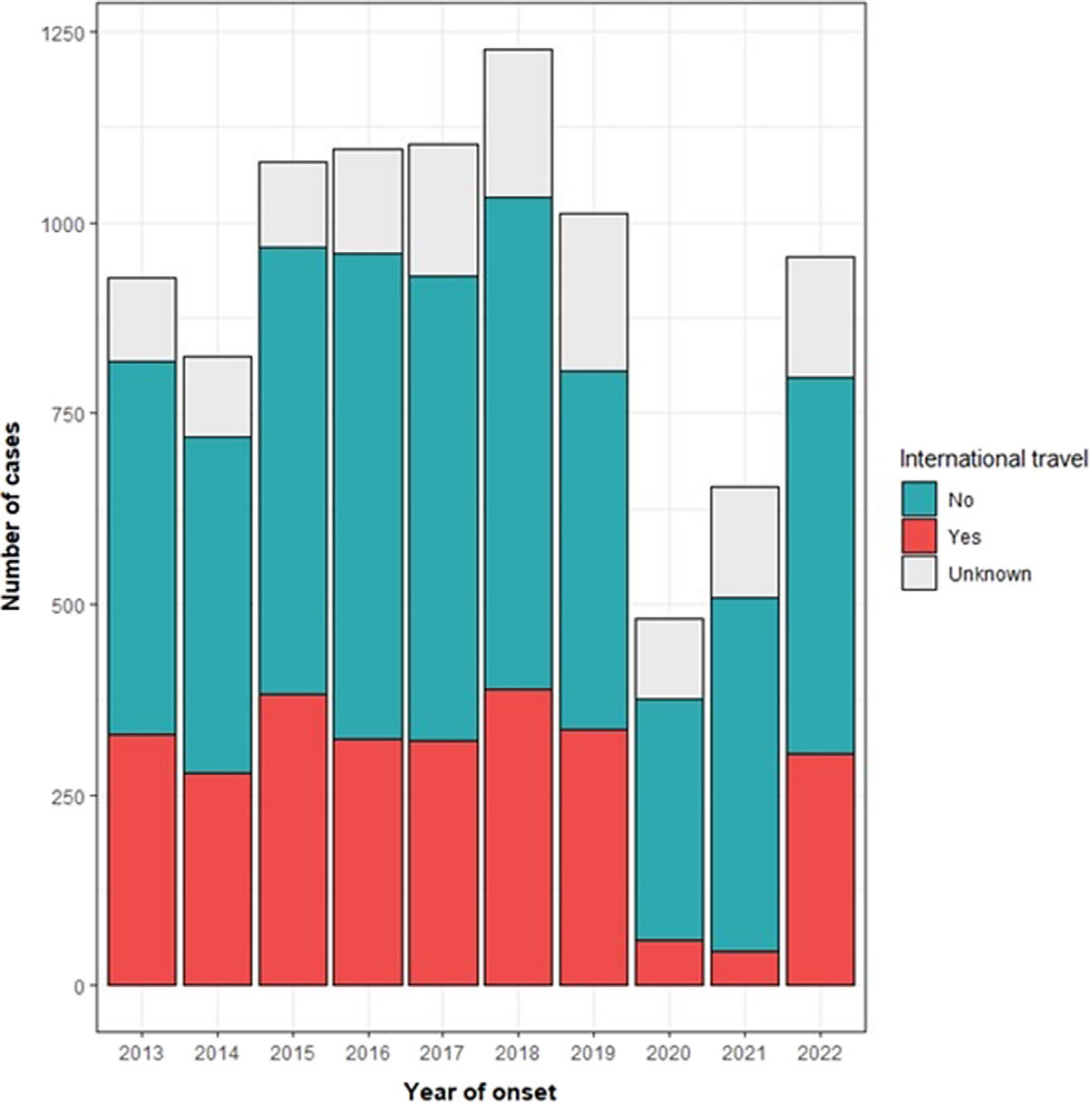
Annual reports of laboratory confirmed gastrointestinal illness* in North East residents indicating number of cases with and without international travel exposures during their incubation period. *Laboratory-confirmed with Cryptosporidium spp, Giardia sp, Hepatitis A, Salmonella spp (typhoidal and non-typhoidal), Shigella spp, Shiga-toxin producing Escherichia coli (STEC; O157 and certain non-O157 serotypes), Vibrio spp and Yersinia spp. During the COVID-19 pandemic, reductions in travel associated infections (59 cases in 2020; −82.5% change; 45 cases in 2021; −86.6% change vs. 337 historic cases; 95% CI: 311–365) were greater than reductions in UK acquired infections (316 in 2020; −42.9% change; 463 cases in 2021; −16.3% change vs. 553 historic cases; 95% CI:494–612).

The proportion of cases associated with international travel remained consistent between 2013 and 2019 (average 38.0%; 95% CI: 35.9–40.1%; range 33.6–41.6%; Chi2: p = 0.96; Figure 1). During England’s COVID-19 pandemic response in 2020 and 2021, total GI infections (travel-associated, UK-acquired, and unknown exposures; n = 480 in 2020 and n = 654 in 2021) were significantly lower than historic figures (2013–2019 average: 1,038; 95% CI: 947–1,129). Reductions in travel-associated infections were greater than reductions in UK-acquired infections (travel-associated infections; −82.5% change in 2020 and − 86.6% in 2021 vs. UK-acquired infections; −42.9% change in 2020 and − 16.3% change in 2021). In 2022, GI infection reports returned to pre-pandemic levels predominantly because of increases in travel-associated cases (total n = 956; travel-associated: n = 303; UK-acquired: n = 493), with the proportion of cases reporting travel comparable to pre-pandemic years (38.1%).

In non-pandemic years, where exposure was known (n = 7,026; 2,660 reporting travel; 37.9%), infections with *Vibrio* species and typhoidal *Salmonella* were exclusively associated with international travel, while around half of infections with hepatitis A, *Shigella spp.*, and non-typhoidal *Salmonella* were travel-acquired (Table 1 and Supplementary Figure 1). Infections caused by *Giardia sp.*, *Cryptosporidium spp.*, and O157 STEC were less commonly associated with travel (31.7%, 28.1%, and 20.8% of infections, respectively). Although average annual numbers of infections associated with travel were relatively low for some pathogens (*Vibrio spp.*: n = <5; typhoidal *Salmonella*: n = 8; Table 1), others contributed considerably to annual GI morbidity in the region (Salmonella: n = 159).

**Table 1.** GI infections reported in NE residents in (2013–2019 average) by pathogen and travel exposure status

				Exposures recorded		International travel reported		UK-acquired	
Pathogen	Exposure duration (days prior to onset)	Average annual number of infections	Annual rate per 100,000 population	Number of cases with travel exposure completed	(% with travel exposure completed)	Number of cases with reported travel	(% cases with reported travel)	Number of UK-acquired infections, with travel exposure completed	(% UK-acquired, with travel exposure completed)
Cryptosporidium spp.	14	299	11.5	263	88.0	74	28.1	189	71.9
Giardia sp.	14	226	8.7	180	79.6	57	31.7	123	68.3
Hepatitis A	8 weeks	8	0.3	<5	50.0	<5	50.0	<5	50.0
Non–typhoidal Salmonella	60	372	14.3	328	88.2	159	48.5	169	51.5
Shigella spp.	7	49	1.9	43	87.8	20	46.3	23	53.5
STEC non–O157	7	16	1.9	7	43.8	<5	28.6	5	71.4
STEC O157	7	49	0.6	48	98.0	10	20.8	38	79.2
Typhoidal Salmonella	7	7	0.3	7	100.0	7	100.0	0	0.0
Vibrio spp.		6	0.2	<5	66.7	<5	100.0	0	0.0
Yersinia spp.	7	5	0.2	<5	80.0	<5	25.0	<5	75.0

Between 2012 and 2019, the percentage of total cases associated with travel remained consistent for most pathogens apart from *Shigella spp.* (Chi2 p = 0.02), where an increase in UK-acquired cases has been observed since 2013, and STEC O157 (Chi2 p = <0.001), where an increase in internationally acquired cases was reported in 2019 (Supplementary Figure 2).

### Demographic characteristics

The demographic characteristics of cases diagnosed with common GI infections following international travel were compared with those of individuals who acquired their infection in the UK (Table 2). The proportions of males (38.5%) and females (37.2%) reporting travel were similar (p = 0.27). The percentage of infections acquired in the UK was significantly higher than infections associated with travel for all age groups; however, children aged under 9 years and adults aged over 60 years were significantly more likely to have acquired their infection in the UK (Table 2, Supplementary Figure 3, and Supplementary Table 1). Ethnicity was poorly completed; however, where available, individuals of Asian ethnicity were more likely to have acquired their infection during international travel (acquired abroad: 66.7%, n = 114 vs. 33.3%, n = 57 acquired in the UK), with the rate of reported travel-associated infection in those of Asian ethnicity (152.8; 95% CI: 126.1–183.6) significantly higher than the rate for those of White ethnicity (59.1; 95% CI: 56.1–62.2).

**Table 2.** Demographic characteristics of NE residents diagnosed with GI infections between 2013 and 2019 with travel exposure information available

			International travel reported		UK-acquired			
Demographic characteristic		Number of cases with travel exposure completed	Number of cases with reported travel	(% cases with reported travel)	Number of UK-acquired infections, with travel exposure completed	(% UK-acquired, with travel exposure completed)	Prevalence ratio	P value
Sex	Male	3,391	1,306	38.5	2085	61.5	0.63	0.27
	Female	3,635	1,354	37.2	2,281	62.8	0.59	
	0–9 years	1,600	443	27.7	1,157	72.3	0.38	
Age group	10–19 years	555	241	43.4	314	56.6	0.77	
	20–29 years	1,067	459	43.0	608	57.0	0.75	
	30–39 years	1,127	449	39.8	678	60.2	0.66	<0.001
	40–49 years	841	358	42.6	483	57.4	0.74	
	50–59 years	818	365	44.6	453	55.4	0.81	
	>60 years	1,018	345	33.9	673	66.1	0.51	
Ethnicity	Asian	171	114	66.7	57	33.3	2.00	
	Black	23	10	43.5	13	56.5	0.77	
	Mixed	42	18	42.9	24	57.1	0.75	<0.001
	Other	30	14	46.7	16	53.3	0.88	
	White	3,753	1,463	39.0	2,290	61.0	0.64	
Index of multiple deprivation	1	1,125	376	30.4	749	66.6	0.50	
	2	861	259	30.1	602	69.9	0.43	
	3	757	286	37.8	471	62.2	0.61	
	4	699	250	35.8	449	64.2	0.56	
	5	505	196	38.8	309	61.2	0.63	<0.001
	6	401	152	37.9	249	62.1	0.61	
	7	484	196	40.5	288	59.5	0.68	
	8	498	229	46.0	269	54.0	0.85	
	9	508	231	45.5	277	54.5	0.83	
	10	392	182	46.4	210	53.6	0.87	
Rural–urban residence	Rural	1,063	363	34.1	700	65.9	0.52	0.007
	Urban	5,167	1994	38.5	3,173	61.5	0.63	
Duration of illness	Median (IQR)	8 days (7)	10 days (7)		8 days (6)			<0.001
Hospital admission	Yes	1,131	370	32.7	761	67.3	0.49	<0.001
	No	4,575	1760	38.5	2,815	61.5	0.63	

### Temporal distribution of travel-associated cases

**Figure 2.**
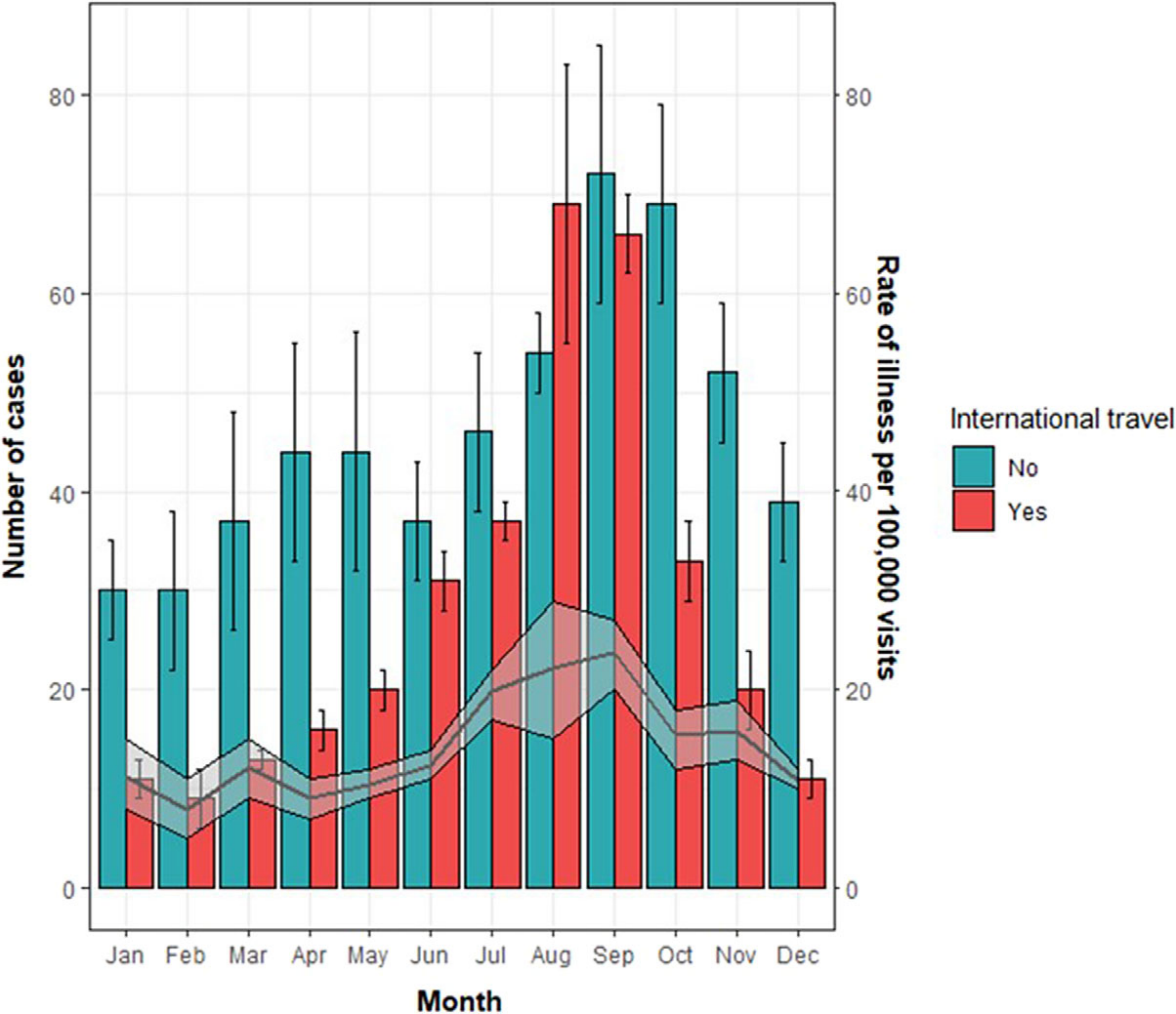
Travel and UK acquired cases* by month of reporting (non-pandemic years) and rate of illness by reported visits (2013–2019) for cases with travel exposure information available. *Laboratory-confirmed with Cryptosporidium spp, Giardia sp, Hepatitis A, Salmonella spp (typhoidal and non-typhoidal), Shigella spp, Shiga-toxin producing Escherichia coli (STEC; O157 and certain non-O157 serotypes), Vibrio spp and Yersinia spp.

Travel-associated cases were highest in the summer with average reported cases in August and September significantly higher than other months (Figure 2). The number of travel-associated cases was significantly lower than the number of UK-acquired cases for all months except between June and September. The monthly distribution of cases was dependent on the geographical region of travel (Supplementary Figure 4). There was less variability in the monthly number of UK-acquired cases; however, the number of cases reported in September and October was significantly higher than the numbers reported in other months. Travel-associated cases corresponded with visits abroad, which were highest in August (333,054 visits; 95% CI: 282,456–383,652) and September (290,153 visits; 95% CI: 241,662–338,643). However, when taking visits into account, rates of illness per 100,000 visits remained highest in August (20.8) and September (22.9) and were lowest in February (8.0).

### Destination of travel

Between 2013 and 2019, 2,357 cases had a country of travel reported (100.0% of cases reported between 2013 and 2019). Of these, 2,284 reported travel to a single country (96.9%). The most common destination country reported by cases was Spain (n = 510), followed by Turkey (n = 322), India (n = 145), and Egypt (n = 131). However, 47.0% of cases reported travel to one of these four countries (n = 1,108). Between 2013 and 2019, Spain (including the Balearic Islands) was the most frequently visited destination for NE England residents with an estimated 4,548,582 visits (Tables 4 and 5) made over the period (649,797 average annual visits; Supplementary Figure 5). France (1,226,916 total and 175,274 average annual visits), the Canary Islands (1,109,696 total and 158,528 average annual visits), and the USA (771,945 total and 110,278 average annual visits) were also common destinations. All destinations with over 100,000 average annual visits were within Europe or the USA.

Rates of illness per 100,000 visits across the period were highest in travellers who visited Africa (107.8 per 100,000 visits; 311 cases) and Asia (61.1 per 100,000 visits; 441 cases) and lowest in travellers visiting European countries (excluding the UK; 9.4 per 100,000 visits; 1,149 cases). Rates of hepatitis A and typhoidal Salmonella were highest in travellers to Asia, and rates of vibrio were comparable for travellers to both Africa and Asia (Table 3). Rates for all other pathogens were highest in travellers returning from Africa. The likelihood of acquiring Shigella in travellers to Africa was 109 times higher than in travellers to Europe, while the rate of acquiring non-typhoidal Salmonella was 527 times higher in travellers to Asia when compared to travellers to Europe (Table 3).

**Table 3.** Rates of illness per 100,000 visits by pathogen and geographical region of travel

	Europe		Africa		America and Caribbean		Asia		Middle East		Rest of world	
Pathogen	Rate	RR	Rate	RR	Rate	RR	Rate	RR	Rate	RR	Rate	RR
Cryptosporidium	3.1	**ref**	18.4	6.0	3.9	1.3	5.0	1.6	2.5	0.8	0.8	0.3
Giardia	1.4	**ref**	15.6	11.4	4.9	3.6	13.2	9.6	1.6	1.2	5.6	4.1
Hepatitis A	0.1	**ref**	—	—	0.2	2.6	0.7	9.9	—	—	—	—
Salmonella	4.3	**ref**	56.8	13.2	12.2	2.8	29.3	6.8	8.3	1.9	8.1	1.9
Shigella	0.1	**ref**	14.2	109.2	1.4	10.5	8.2	62.9	—	—	2.01	15.5
STEC non–O157	0.07	**ref**	1.4	19.9	—	—	—	—	—	—	0.4	5.7
STEC O157	0.4	**ref**	3.1	7.4	0.3	0.6	0.3	0.7	0.63	1.5	0.4	1.0
Typhoidal Salmonella	0.01	**ref**	0.4	35.0	0.2	18.0	5.3	527.0	0.63	63.0	—	—
Vibrio	0.02	**ref**	2.1	104.0	0.3	13.5	2.2	111.0	—	—	0.4	20.0
Yersinia	0.02	**ref**	0.7	34.5	—	—	0.1	7.0	0.32	16.0	—	—

*Note:* Rates per 100,000 visits were calculated using the total number of visits to each country or country group between 2013 and 2019 calculated using the ‘Final weight’ variable in the Travelpac data set for 2013–2019 and the total number of cases reporting travel to the location between 2013 and 2019.

Of the 20 countries reporting a rate of illness of over 100 cases per 100,000 visits (classified here as high risk), only Turkey (147.9 per 100,000), India (110.6 per 100,000), and Tunisia (101.5 per 100,000) had more than 10,000 visitors annually (Table 4). Of note, high rates of illness were also associated with tourist destinations such as Egypt (386.4 per 100,000 visits) and the Dominican Republic (244.2 per 100,000 visits), which receive fewer than 10,000 visitors annually but like Turkey are also popular tourist destinations. The highest rate of illness was reported from travellers to Nepal (769.4 per 100,000 visits), but less than 250 NE residents were estimated to visit Nepal each year. Rates of illness were high from countries in South Asia and Africa, including Kenya (400.9 per 100,000), Pakistan (252.0 per 100,000), and Cambodia (113.7 per 100,000). Several countries in South and Central America also had high rates of illness per 100,000 visits (Colombia: 208.6; Ecuador: 169.5; and Peru: 139.1).

**Table 4.** Total cases per 100,000 visits by destination country indicating the average annual number of visitors per country

Cases per 100,000 visits	Destination country
<10	Australia, Austria, Belgium, Canary Islands, China (excluding Taiwan)/Tibet, Croatia, Czech Republic, France/Corsica, Germany, Holland, Hungary, Iceland, Irish Republic, Israel, Italy/Sardinia, Kuwait, Latvia, Madeira/Azores, Malaysia, New Zealand, Norway, Poland, Portugal, Romania, Switzerland, USA
10.1–20	Greece/Crete/Rhodes, Hong Kong, Iran, Japan, Kazakhstan, the Maldives, Russia, Slovakia, South Africa, Spain, Trinidad and Tobago, the United Arab Emirates
20.1–30	Argentina, Bolivia, Brazil, Cyprus, the Democratic Republic of Congo, Lebanon, Libya, Malta, Mauritius, Nigeria, Philippines, Qatar
30.1–40	Azerbaijan, Barbados, Bulgaria, Iraq, Jordan, Nevis/St. Kitts, Singapore
40.1–50	Gambia, Jamaica, Mongolia, Saudi Arabia, Sri Lanka
50.1–60	Costa Rica, Montenegro, Thailand
60.1–70	Mexico, Morocco, Serbia, Vietnam
70.1–80	Antigua, Malawi, Uzbekistan
80.1–90	Bangladesh, Cape Verde Islands
90.1–100	Bali/Borneo/Indonesia, Cuba, Uganda, Zambia
100.1–200	Afghanistan, Burkina Faso, Cambodia/Kampuchea, Ecuador, Equatorial Guinea, Ghana, India, Namibia, Peru, Tanzania, Tunisia, Turkey
200.1–300	Colombia, the Dominican Republic, Ethiopia, Madagascar, Pakistan
>300.1	Egypt, Kenya, Nepal, North Sudan, Somalia

*Note:* Rates per 100,000 visits were calculated using the total number of visits to each country or country group between 2013 and 2019 calculated using the ‘Final weight’ variable in the Travelpac data set for 2013–2019 and the total number of cases reporting travel to the location between 2013 and 2019.

Countries in blue have <10,000 visitors annually, those in purple have between 10,000 and 50,000 visits annually, and those in red have more than 50,000 visits annually.

**Table 5.** Rate ratios for travel destinations compared to Spain (reference country) indicating the average annual number of visitors per country

RR	Destination country
< 1.00	Australia, Austria, Belgium, Canary Islands, China (excluding Taiwan)/Tibet, Croatia, Czech Republic, France/Corsica, Germany, Holland, Hungary, Iceland, Irish Republic, Israel, Italy/Sardinia, Kuwait, Latvia, Madeira/Azores, Malaysia, New Zealand, Norway, Poland, Portugal, Romania, Switzerland, USA
1.00	Spain (reference)
1.01–2.00	Greece/Crete/Rhodes, Hong Kong, Iran, Japan, Kazakhstan, the Maldives, Russia, Slovakia, South Africa, Trinidad and Tobago, the United Arab Emirates
2.01–3.00	Argentina, Bolivia, Brazil, Cyprus, the Democratic Republic of Congo, Lebanon, Libya, Malta, Mauritius, Nigeria, Philippines, Qatar, Singapore
3.01–4.00	Azerbaijan, Barbados, Bulgaria, Iraq, Jordan, Mongolia, Nevis/St. Kitts
4.01–5.00	Gambia, Jamaica, Saudi Arabia, Sri Lanka
5.01–6.00	Costa Rica, Montenegro, Thailand, Vietnam
6.01–7.00	Mexico, Morocco, Serbia
7.01–8.00	Antigua, Cape Verde Islands, Malawi, Uzbekistan
8.01–9.00	Bali/Borneo/Indonesia, Bangladesh, Uganda
9.01–10.00	Cuba, Tunisia, Zambia
10.01–20.00	Afghanistan, Burkina Faso, Cambodia/Kampuchea, Ecuador, Equatorial Guinea, Ghana, India, Namibia, Peru, Tanzania, Turkey
20.01–30.00	Colombia, the Dominican Republic, Ethiopia, Madagascar, Pakistan
30.01–40.00	Egypt, Kenya, Somalia
>40.01	Nepal, North Sudan

*Note:* Rates per 100,000 visits were calculated using the total number of visits to each country or country group between 2013 and 2019 calculated using the ‘Final weight’ variable in the Travelpac data set for 2013–2019 and the total number of cases reporting travel to the location between 2013 and 2019. Rates were compared to a reference country (Spain), which was the most common destination of travel for NE residents between 2013 and 2019.

Countries in blue have <10,000 visitors annually, those in purple have between 10,000 and 50,000 visits annually, and those in red have more than 50,000 visits annually.

Of the 2,404 individuals with routinely collected exposure data, it was possible to identify the type of accommodation used while travelling for 1,868 cases (77.7%). However, 92.5% of cases visiting Europe, 86.2% visiting the Americas, 84.7% visiting Africa, and 83.8% visiting Asia stayed in hotels. Staying with family and friends while travelling was less commonly reported; 5% of cases reported travelling to Africa, 4.2% of cases reported travelling to Asia, 3% of cases reported travelling to the Americas, and 2.1% of cases reported travelling to Europe. A total of 1,233 cases reported named premises, of which 1,058 were unique and were only reported by one case (85.8%). The remaining premises were associated with clusters of between 2 and 13 cases (median: 2, interquartile range (IQR): 1).

Clusters, defined as two or more cases, were most commonly associated with Salmonella (n = 54) and *Cryptosporidium spp.* (n = 41), fewer than 10 clusters were reported for each of Giardia, Shigella, or STEC (O157) or STEC (non-0157). Salmonella outbreaks were predominantly associated with travel to Turkey (n = 19 clusters, n = 42 cases), Egypt (n = 11 clusters; n = 23 cases), and Mexico (n = 6; 13 cases). Cryptosporidium outbreaks were predominantly associated with Spain (n = 17; 39 cases), Turkey (n = 6; n = 20 cases), the Canary Islands (n = 4; n = 16 cases), and Egypt (n = 4; n = 9 cases). Overall, eleven hotels had clusters reported in two separate years and four hotels reported clusters in three separate years.

## Discussion

Through this analysis of laboratory and exposure data for cases of notifiable GI infections in NE England, we show that international travel is a major risk factor, contributing substantially to the burden of infection in the region. Furthermore, as there has been no reduction in the proportion of travel-associated infections in non-pandemic years since 2013 this work highlights the need to better understand the risk factors associated with developing GI illness while travelling.

The considerable decline in GI infections observed during the COVID-19 pandemic was likely driven by a reduction in travel-associated infections. This suggests the overall burden of GI illness could be reduced if improvements were made to the number of individuals acquiring an illness while travelling abroad, particularly as returning travellers may be seeding illness and ongoing transmission across the wider population within the UK [13]. Pathogen-specific reductions in GI infections were also observed in England overall during the COVID-19 pandemic, with diagnoses of pathogens such as Salmonella and Cryptosporidium, which are commonly associated with foreign travel, remaining lower than infections with pathogens such as STEC, which are often UK-acquired [13, 14].

The strength of this study is that it used denominator data for international travel for the NE England population allowing rates to be determined. Country-specific case numbers may correlate with the volume of travel to a destination, which makes it challenging to draw conclusions on the destination-specific risks. For example, Spain was the most commonly reported travel destination of cases, but it was also the most common destination of travel for NE England residents, with the rate of illness per visit similar to that reported for other European countries. Conversely, travel to countries in Africa and Asia was less common for NE England residents, but it was associated with a high risk of illness. With globalization, changes in travel patterns, and an increasing non-UK-born population in NE England, it is possible that visits to high-risk destinations will increase [15].

Travelling to high-risk countries to visit friends and relatives is a known risk factor for GI infections [16], with 75% of enteric fever cases occurred in individuals travelling to visit friends and relatives and high rates observed among individuals of Pakistani or South Asian ethnicity [17]. In our study, where ethnicity was completed, those of Asian ethnicity were more likely to have acquired their infection during international travel, with the rate of international travel associated with Asian ethnicity significantly higher than for those of white ethnicity. Due to small numbers, there were insufficient data available to demonstrate that higher rates of illness in those of Asian ethnicity were the result of travel to visit friends and relatives, but the study did demonstrate that a higher proportion of cases reported as visiting countries in Asia were staying with friends or family. However, it has also been shown that residents from ethnic minorities in high-income countries have lower health literacy with language proficiency and lower social support identified as key barriers [18]. Future work looking at infections across England overall could provide further evidence as to why rates of illness are higher in those of Asian ethnicity.

While the findings of this study do not indicate absolute risk associated with travel to specific areas, they do allow for comparisons in patterns of illness between countries. High rates of illness were reported following travel to countries or regions, which were documented in other studies and in travel guidance to be ‘high risk’ for travel-associated GI infections [3, 4, 19]. This study additionally highlights increased rates of illness associated with ‘all-inclusive’ holiday destinations including the Dominican Republic, Turkey, and Egypt, with rates per 100,000 visits as high as destinations commonly categorized as ‘high risk’ [12]. This has also been reported in other studies with the Dominican Republic shown to have the third highest number of all-pathogen travel-related diagnoses in returning travellers reported in the U.S. GeoSentinel Network between 2012 and 2021, after Mexico and India [19]. All-inclusive travel to low- and middle-income countries may be perceived as lower risk as this type of travel and companies offering it are often mainly associated with lower-risk destinations such as high-income countries in Western Europe. Higher rates of illness reported from Turkey and Egypt may also be associated with outbreak activity at hotel resorts. Over the period, 175 hotels were associated with more than one case with clusters more commonly reported in travellers to Turkey and Egypt.

As travel-associated infections are only included if diagnosed following return to NE England, this may lead to an underestimation of infections, particularly those that may be short-lived or less severe [20]. Conversely, there may be an overestimation of travel as a cause of illness with primary care physicians often more often arranging stool testing for individuals reporting international travel than for those with similar symptoms without a history of travel [21]. It has also been shown previously that travel as a risk factor may be overestimated, with cases associated with domestic transmission misclassified as travel-associated when shorter incubation period durations are taken into account [22]. A further limitation is that denominators are estimates based on survey data and may not fully reflect travel patterns of NE England residents.

This study highlights that international travel remains a common risk factor for enteric infections. However, it was not possible to explore in detail the risks while travelling using secondary analysis of routinely collected data due to the unstructured nature of data collected. Given the large proportion of diagnosed cases acquiring their infection abroad, we recommend that further studies be undertaken to collect structured travel-specific data from cases diagnosed with GI infections following travel, and that this be considered within routine surveillance, to help inform public health messages aimed at the prevention and reduction of travel-associated GI illness in travellers.

## Supporting information

Love et al. supplementary material Love et al. supplementary material

## Supplementary material

For supplementary material accompanying this paper visit http://doi.org/10.1017/S0950268824000827. click here to view supplementary material
