# Supplementary material for: International travel as a risk factor for gastrointestinal infections in residents of North East England
Source: Epidemiol Infect. 2024 May 27;152:e97. doi: 10.1017/S0950268824000827 (PMC11736447; doi:10.1017/S0950268824000827)

**Supplementary Figure 1 – Number of cases reported between 2013-2019 by pathogen and travel exposure status with percentages indicating those with an exposure reporting travel**


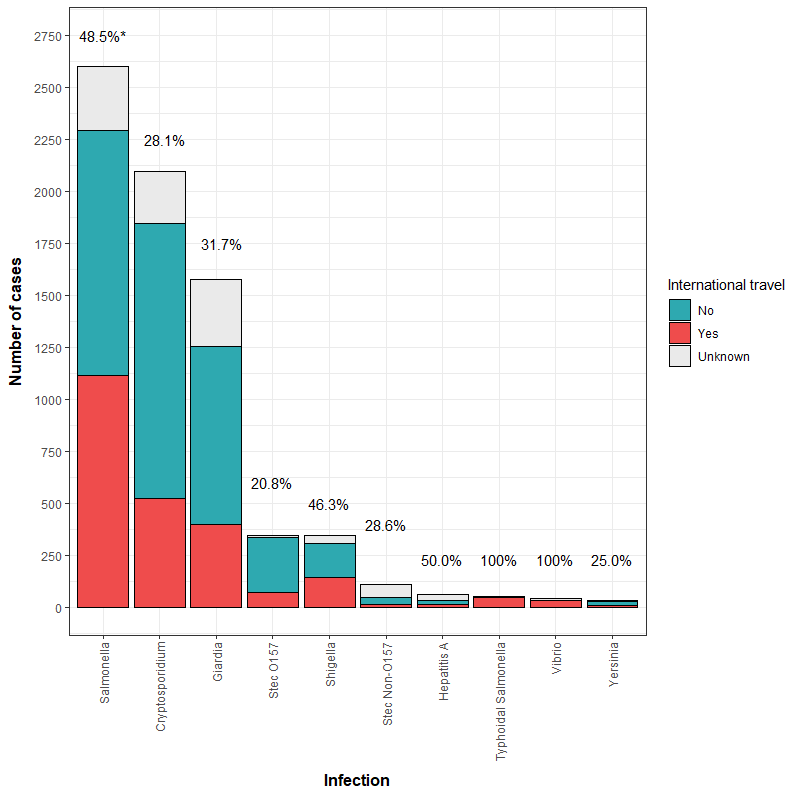


* Percentage indicates those with a response who reported international travel during their incubation period.

**Supplementary Figure 2 – Percentage of cases with international travel acquired or UK acquired infections* by pathogen and year (2013-2022).**


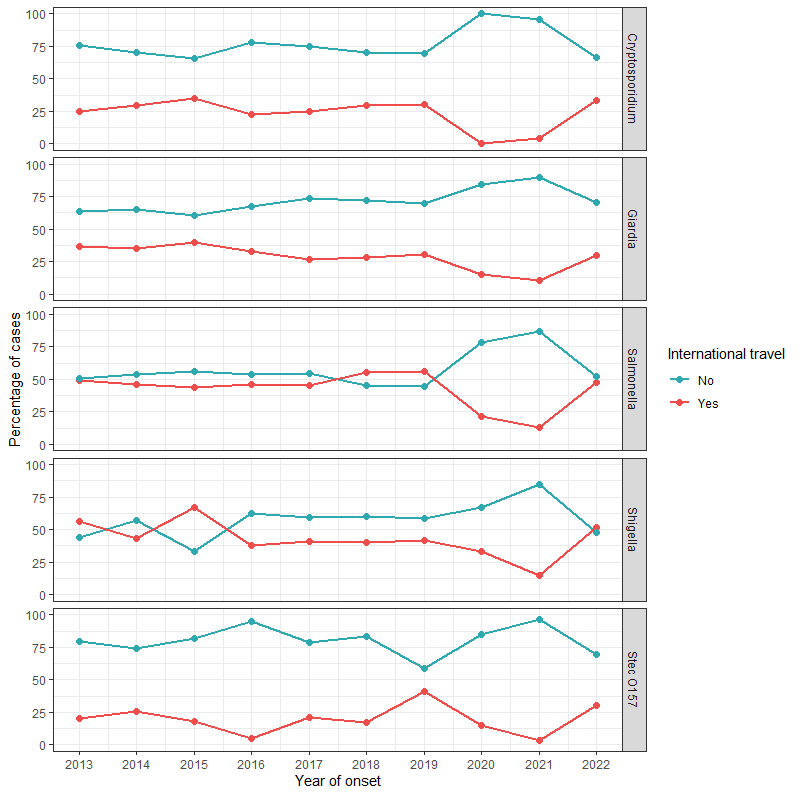


* Excludes those where travel exposure status is not recorded. Hepatitis A, *Yersinia* spp and *Vibrio* spp excluded due to small numbers

Chi2 for 2013-2019. Cryptosporidium p=0.67; Giardia p=0.69; Salmonella p=0.83; Shigella p=0.02 and STEC O157 p=<0.001

**Supplementary Figure 3 – Proportion of North East cases diagnosed with gastrointestinal illness reported between 2013 and 2019 with known exposures by age group and travel status**


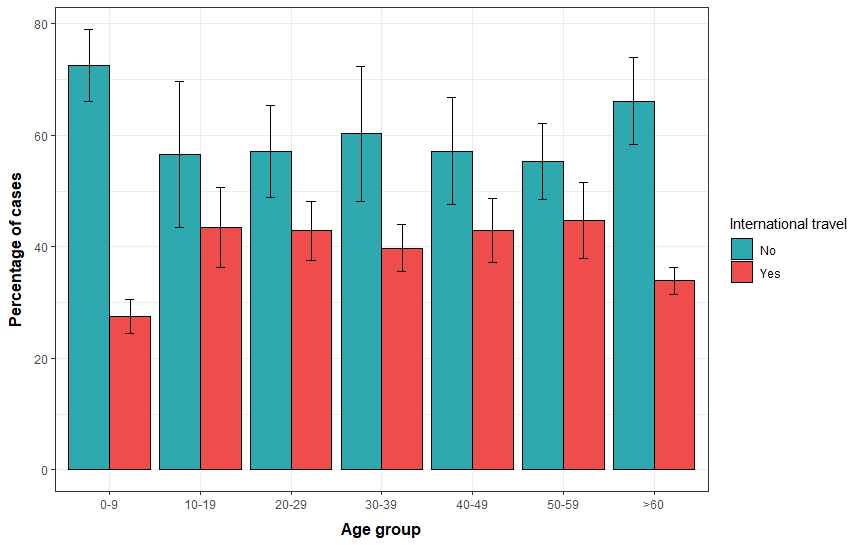


**Supplementary Table 1 – Direct standardised rates for age group and ethnicity group per 100,000 North East residents**

| **Number of cases with travel exposure completed** | **Rate of cases with reported travel per 100,000 population (95% CI)** | | **Rate of UK acquired cases, with travel exposure completed per 100,000 population (95% CI)** |
| --- | --- | --- | --- |
| 0 – 9 years | | 18.8 (14.2 - 24.5) | 49.2 (41.5 - 57.9) |
| 10 – 19 years | | 9.99 (6.75 - 14.3) | 13.0 (9.26 - 17.8) |
| 20 – 29 years | | 16.1 (12.2 - 20.9) | 21.3 (16.8 - 26.7) |
| 30 – 39 years | | 16.8 (12.7 - 21.8) | 25.4 (20.3 - 31.4) |
| 40 – 49 years | | 14.4 (10.5 - 19.3) | 19.5 (14.9 - 25.0) |
| 50 – 59 years | | 12.2 (8.90 - 16.3) | 15.1 (11.4 -19.6) |
| >60 years | | 6.07 (4.39 - 8.17) | 11.8 (9.44 - 14.7) |
| Asian | | 153 (126 - 184) | 76.4 (57.9 - 99.0) |
| Black | | 75.6 (36.2 - 139) | 98.3 (52.3 - 168) |
| Mixed | | 80.2 (47.5 - 127) | 107 (68.5-159) |
| Other | | 127 (69.2 - 213) | 145 (82.7-235) |
| White | | 59.1 (56.1 - 62.2) | 92.5 (88.8 - 96.4) |

Data completeness for age: 100%. Data completeness for ethnicity: 55% for UK acquired cases and 60.9% for travel associated cases. 1,966 unknown ethnicity in UK acquired and 1,041 unknown ethnicity in travel associated.

**Supplementary Figure 4 – North East cases diagnosed with gastrointestinal illness reported between 2013 and 2019 with travel exposures by month of report and geographical area of travel**


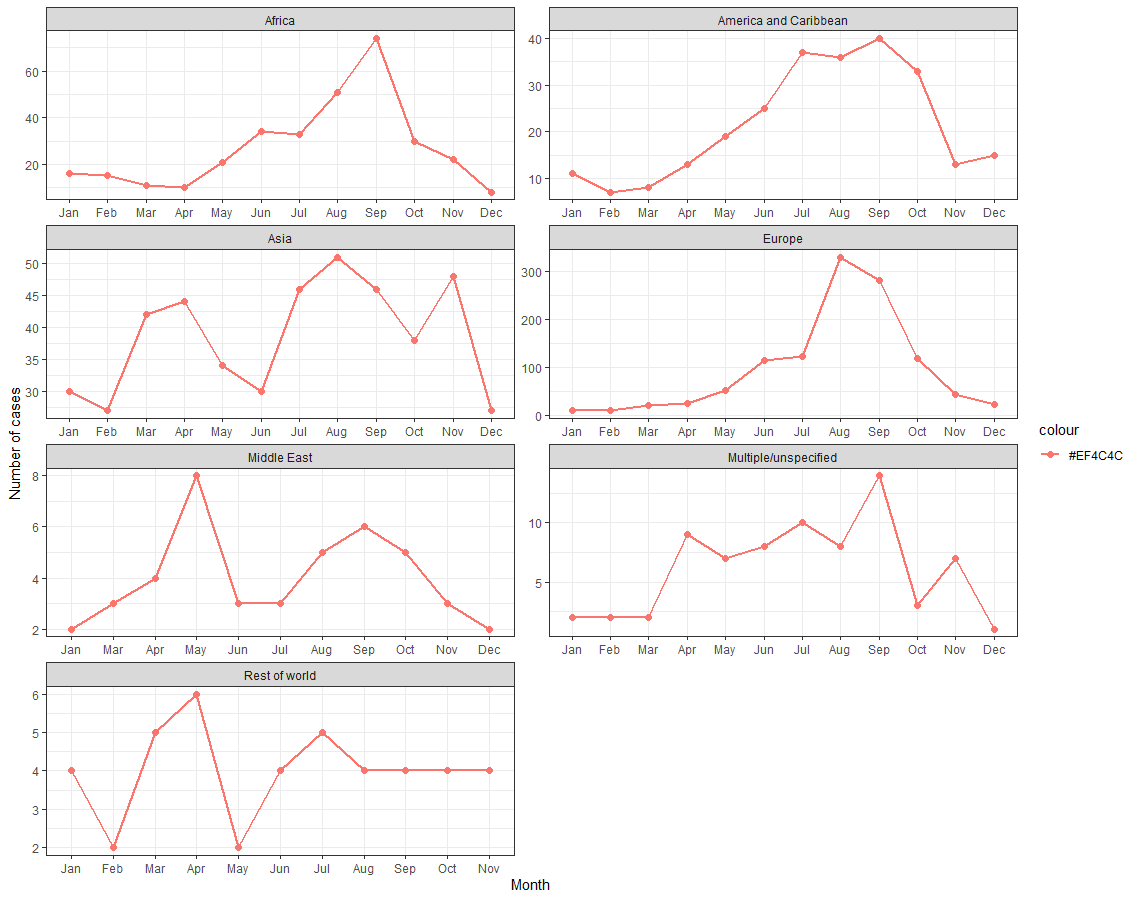


**Supplementary Figure 5 – Frequency of visits by North East residents estimated using the ONS passenger survey showing destinations with a) <10,000 annual visits, b) 10,000 to 50,000 annual visits and c) > 50,000 visits**


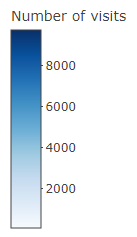

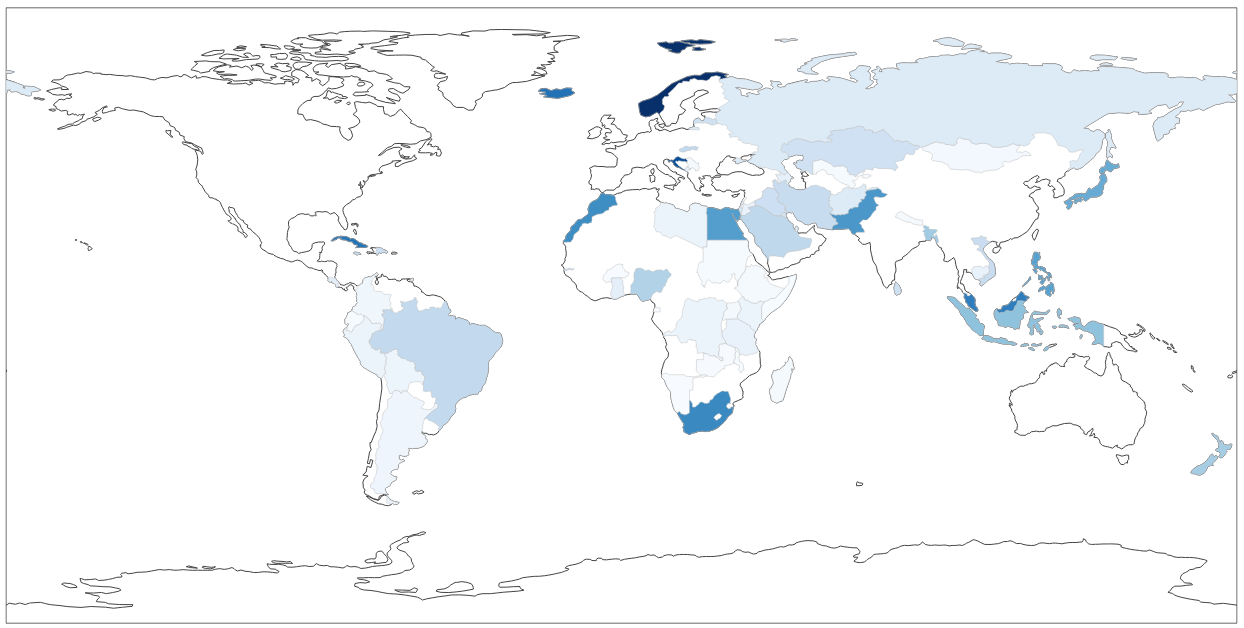


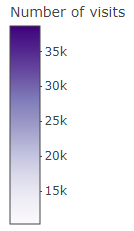

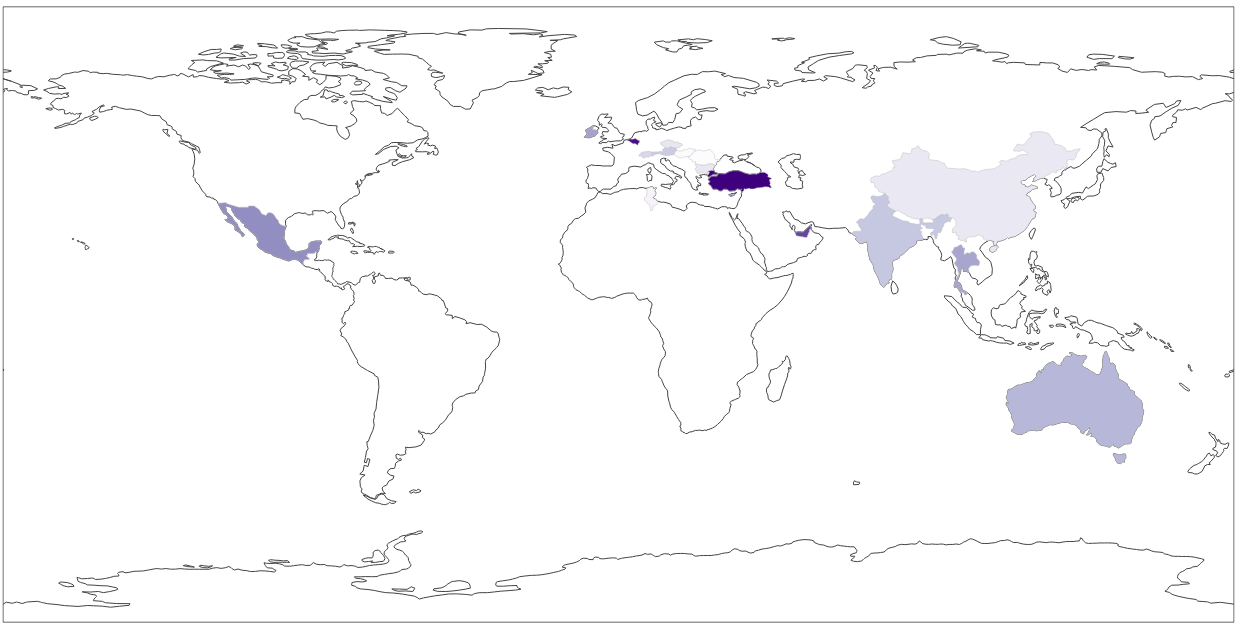


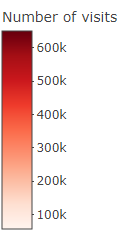

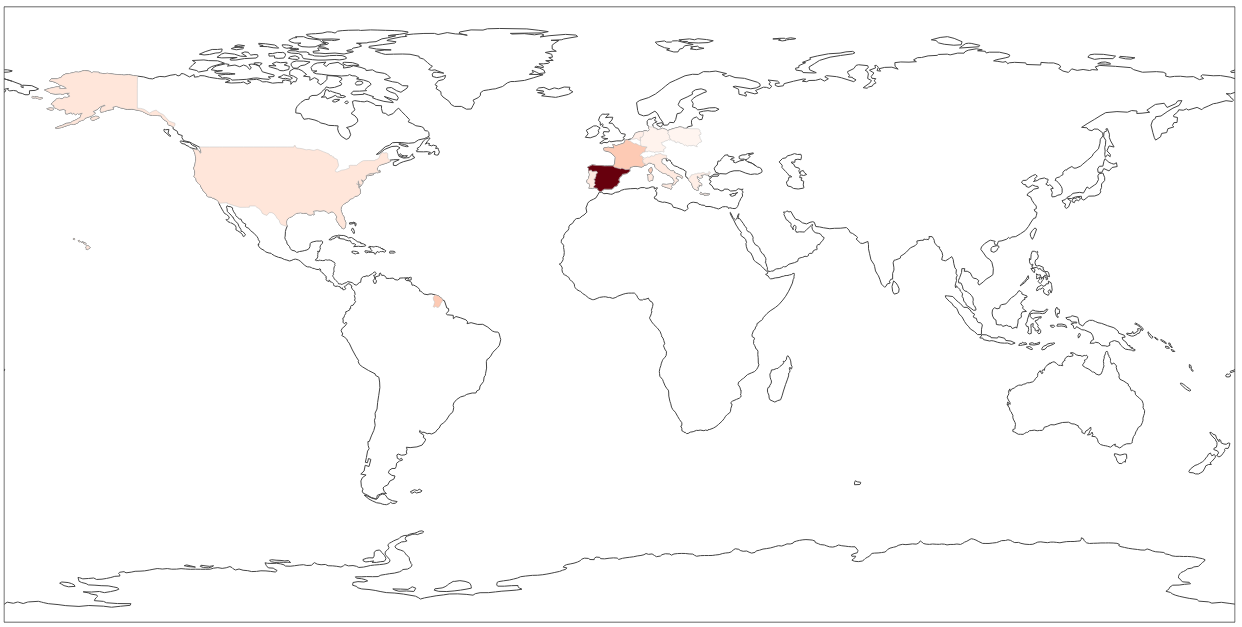

Supplement: Love et al. supplementary material [file S0950268824000827sup001.docx]
